# Prevalence of antibiotic resistance genes its association with microbiota in raw milk of northwest Xinjiang

**DOI:** 10.3389/fmicb.2025.1595051

**Published:** 2025-07-11

**Authors:** Yan Zhao, Yifan Niu, Min Zhao, Wanting Huang, Yanan Qin

**Affiliations:** ^1^College of Smart Agriculture (Research Institute), Xinjiang University, Urumqi, China; ^2^Xinjiang Key Laboratory of Biological Resources and Genetic Engineering, College of Life Science & Technology, Urumqi, Xinjiang, China

**Keywords:** antibiotic resistance genes, antibiotic-resistant bacteria, host, High throughput qPCR, raw milk

## Abstract

The issue of antibiotic resistance caused by antibiotic resistance genes (ARGs) has become a significant concern in environmental research in recent years, while raw milk is an important link in the food chain and has become one of the carriers and reservoirs of ARGs, which has not been taken seriously. This research employed high-throughput quantitative PCR and Illumina sequencing techniques targeting the 16S rRNA gene. These methods were used to examine the bacterial community composition and genes associated with antibiotic resistance in raw milk samples collected from the northwestern area of Xinjiang. An aggregate of 31 distinct resistance alleles were identified, with their abundance reaching as high as 3.70 × 10^5^ copies per gram in the analyzed raw milk samples. Microorganisms harboring ARGs that confer resistance to beta-lactams, tetracyclines, aminoglycosides, and chloramphenicol derivatives were prevalent in raw milk. Procrustes analysis revealed a certain degree of correlation between the microbial community and the antibiotic resistance gene (ARG) profiles. Furthermore, network analysis demonstrated that *Actinobacteria* and *Firmicutes* were the predominant phyla exhibiting co-occurrence relationships with specific ARGs. Combining the findings from Variance Partitioning Analysis (VPA), the distribution of ARGs was mainly driven by three factors: the combined effect of physicochemical properties and mobile genetic elements (MGEs) (33.5%), the interplay between physicochemical parameters and microbial communities (31.8%), and the independent contribution of physicochemical factors (20.7%). The study demonstrates that the overall abundance of ARGs correlates with physicochemical parameters, bacterial community composition, and the presence of MGEs. Furthermore, understanding these associations facilitates the evaluation of antibiotic resistance risks, thereby contributing to enhanced farm management practices and the assurance of food safety.

## Introduction

The emergence and dissemination of antibiotic resistance genes (ARGs) represent a critical global health challenge, undermining the efficacy of antibiotics in human and veterinary medicine (Cook and Wright, 2022). Antimicrobial resistance (AMR) driven by ARGs poses multidimensional threats to public health, environmental safety, and food security (Bonetta et al., 2023). Food systems, particularly livestock farming, serve as significant reservoirs for ARGs. The use of antibiotics in animal husbandry increases selective pressure, promoting the survival of antibiotic-resistant bacteria that can release ARGs into the surrounding environment. The overuse of antibiotics for therapeutic and growth-promoting purposes fosters an environment conducive to the proliferation of antibiotic-resistant bacteria. The resulting manure often contains elevated levels of these resistant bacteria and their associated ARGs. When this manure is applied as fertilizer or inadequately treated prior to use, it may contaminate soil and water sources, thereby impacting nearby dairy farms (Yang et al., 2020). Intensive agricultural practices, including antibiotic overuse in dairy farms, have been linked to the selection and spread of resistant bacteria and ARGs (Jian et al., 2021).

In China, antibiotic consumption remains disproportionately high, with nearly half of annual production used in livestock and poultry sectors (Xu et al., 2024). Regional data from Xinjiang show extensive antibiotic exposure. The detection rate of antibiotic residues in beef and mutton in Xinjiang region is high, highlighting that animal husbandry is the main source of pollution (Zhang et al., 2021). Despite extensive research on ARGs in environmental matrices like soil and water, the prevalence, diversity, and origins of ARGs in raw milk remain inadequately characterized (Xin et al., 2022). This knowledge gap limits our understanding of how ARGs may transfer between environmental bacteria, milk microbiota, and potential human pathogens. Raw milk, a nutrient-rich medium, harbors complex microbial communities and serves as a potential reservoir for ARGs through horizontal gene transfer (HGT) mediated by MGEs such as plasmids and transposons (Wu et al., 2020).

ARG dissemination in raw milk differs fundamentally from antibiotic persistence. While antibiotics exert selective pressure by inhibiting susceptible bacteria (Chait et al., 2016), ARGs spread independently via HGT mechanisms—conjugation (plasmid transfer), transduction (phage-mediated transfer), and transformation (free DNA uptake). For example, biofilms on milking equipment and phages in agricultural waste facilitate ARG transfer to raw milk (Kaszab et al., 2023). Additionally, fecal contamination and suboptimal farm hygiene introduce resistant bacteria into milk, further exacerbating ARG spread (Forster et al., 2022; Tóth et al., 2020). HGT is particularly concerning as it facilitates the transfer of ARGs from environmental or non-pathogenic bacteria to pathogenic bacteria present in milk or dairy products. Storage conditions also modulate ARG dynamics: refrigeration favors psychrotolerant bacteria like *Pseudomonas*, which dominate raw milk microbiota and harbor ARGs (Zhang et al., 2020). Comparisons with European and U.S. dairy systems reveal similar ARG trends (e.g., dominance of β-lactam and tetracycline resistance genes), but Xinjiang's raw milk exhibits higher *vanXD* abundance, likely due to regional antibiotic use patterns (Oliver et al., 2020).

Previous studies have predominantly focused on ARG profiles in wastewater or soil, leaving a gap in understanding ARG-host associations and drivers in raw milk (Kang et al., 2022). This study integrates high-throughput qPCR, 16S rRNA sequencing, and multivariate analyses to investigate ARG diversity, abundance, and drivers in raw milk from Northwest Xinjiang. We hypothesize that ARG distribution is governed by synergistic interactions among physicochemical properties, microbial communities, and MGEs. By identifying key ARG hosts and HGT mechanisms, this work provides critical insights for mitigating AMR risks in dairy systems and informs strategies to enhance food safety.

## Materials and methods

### Samples collection

In August 2021, an assortment of 16 milk specimens, comprising four sets with four duplicates each, was gathered from four expansive dairy operations in Changji, Xinjiang (spanning 87°50′E to 87°80′E and 43°70′N to 45°20′N, populations ≥1,500 for every farm). These farms were selected as representative operations in the region, with management practices that aligned with local norms to ensure the generalizability of findings. From each farm, four raw milk samples (16 total) were aseptically collected from sterile bulk storage tanks immediately after morning milking. To prevent external contamination, all collection tools and containers were pre-sterilized, and operators wore sterile gloves and masks during sampling. Each sample was transferred into a 200-milliliter sterile plastic container, flash-frozen on dry ice within 15 min of collection, and transported to the laboratory under continuous cryogenic conditions (−20°C). Samples were stored at −80°C until DNA extraction.

### Physicochemical parameters analyses

All laboratory procedures, including DNA extraction, were conducted under aseptic conditions to minimize microbial contamination. Physicochemical parameters of raw milk from four farms were carried out. The contents of protein, fat, and non-fat milk solids were measured by milk composition analyzer (Foss 91828605). The physicochemical parameters of each sample were measured independently three times, and the average was taken as the final result. Prior to the experiment, the milk analyzer was calibrated using a standard calibration solution (Foss official calibration kit) to ensure measurement accuracy. All operations are performed by the same experimentalist to eliminate manual differences.

### DNA extraction

DNA extraction was performed by a modified CTAB protocol, which is optimized for liquid substrates (Minas et al., 2011). In simple terms, the microbial cells are centrifuged into balls and cleaved with lysozyme and protease K. DNA purity (A260/A280 >1.8) was verified by NanoDrop ND-1000. To monitor contamination, blank controls (sterile water) and environmental controls were treated in parallel, and no amplification was detected in subsequent PCR analysis. The extracted DNA was diluted to 1 ng/μL in nuclease-free water for downstream analysis.

### High-throughput quantitative PCR and data analysis

This study employed the WaferGen SmartChip Real-time PCR system (Xuanchen Biotechnology Co., Ltd., Xi'an, China) for high-throughput quantitative detection of ARGs in raw milk samples. A total of 348 primer pairs were designed, including 330 targeting ARGs, 17 targeting MGEs, and one pair targeting the 16S rRNA gene as an internal reference (Su et al., 2015). Primer specificity and amplification efficiency were rigorously validated in prior studies: amplification efficiency for each primer pair was required to fall within 90%−110%, and at least two positive technical replicates were required to confirm amplification. The PCR protocol included an initial denaturation at 95°C for 10 min, followed by 40 cycles of 95°C for 30 s and 60°C for 30 s. All samples were analyzed in triplicate, with a cycle threshold (CT) set at 35 to define the detection limit. A gene was considered present only if detected in all three replicates.

Gene quantification utilized two metrics: relative gene copy numbers and normalized ARG abundance per bacterial cell. Relative copy numbers were calculated using the formula 10^(35 − CT)^/(10/3), where CT represents the threshold cycle. To normalize for bacterial cell density, ARG copy numbers were adjusted by dividing the relative ARG copy number by four times the relative 16S rRNA gene copy number, accounting for the average of four 16S rRNA copies per bacterial cell. Bacterial cell counts were estimated by dividing the 16S rRNA copy number by 4, a value derived from the Ribosomal RNA Operon Copy Number Database (rmDB v4.3.3) based on dominant phyla in raw milk. Data analysis was performed automatically using the instrument's qPCR software (version 2.7.0.1), with amplification results retained only if curve-fitting criteria were satisfied.

### High-throughput 16S rRNA gene sequencing

DNA extracted from raw milk samples was purified using the FastDNA SPIN Kit for soil (MPbio, Santa Ana, CA, USA). DNA quality and concentration were assessed via a spectrophotometer (ND−8000, Thermo Fisher Scientific, Waltham, MA, USA) and 1.5% agarose gel electrophoresis. The hypervariable V3–V4 regions of the bacterial 16S rRNA gene were amplified using barcoded primers. Amplified products were verified by 2% agarose gel electrophoresis, and libraries were constructed using the TruSeq^®^ DNA PCR-Free Sample Preparation Kit. Library concentrations were quantified using Qubit and qPCR, followed by paired-end sequencing on the Illumina NovaSeq6000 platform (BIOTREE, Shanghai, China).

Raw sequencing reads were processed using FLASH (v1.2.7) to merge paired-end reads, followed by filtering of adapters, low-quality sequences, and chimeras to generate high-quality tags. Operational taxonomic units (OTUs) were clustered at 97% similarity using the Uparse algorithm (v7.0.1001), with the most abundant sequence selected as the representative OTU sequence. Taxonomic classification was performed using the Mothur method (v1.8.0) against the SILVA SSUrRNA database (SILVA138) with a confidence threshold of 0.8–1. Unclassified OTUs were separately annotated. Sequence alignment was conducted using MUSCLE (v3.8.31), and alpha diversity indices (Chao1, Shannon, Simpson, ACE) were calculated using QIIME 2 (v2023.9). Beta diversity was assessed using Bray–Curtis distances, and principal coordinate analysis (PCoA) with Tukey tests was applied to evaluate differences in microbial community structures across samples.

### Statistical analysis

Statistical analyses were selected based on their suitability for microbial community and ARG profile data. Data normalization and averaging were performed in Microsoft Excel 2021 (Microsoft, Redmond, WA, USA). Alpha diversity indices (Shannon, Chao1) were computed via QIIME 2 software version 2023.9 (QIIME development team, Aurora, CO, USA). Beta diversity analysis utilized Bray–Curtis dissimilarity, which is robust for compositional microbiome data. Principal coordinate analysis (PCoA) and Procrustes tests (R v4.3.1) were applied to assess ARG-microbiota correlations. Redundancy analysis (RDA) and Variance Partitioning Analysis (VPA) were performed using Canoco 5.15 and the Majorbio Cloud Platform, respectively, to evaluate the effects of physicochemical factors, microbial communities, and MGEs on ARG distribution. Mantel and Adonis tests (9999 permutations) were used to validate spatial and categorical associations. Conduct network analysis (using the OmicStudio tool) to investigate the co-occurrence patterns of ARGs and bacterial phyla based on Spearman's rank correlation. Origin 2021 (OriginLab, Northampton, Massachusetts, USA) was used to construct bar charts.

## Results and discussion

### Physicochemical parameters of raw milk

The average contents of protein, fat, and non-fat milk solids of milk samples (Table 1) were 3.10–3.17%, 3.54% to 3.93%, 8.73% to 8.83% respectively. The physicochemical parameters of raw milk are influenced by factors such as breed, stage of lactation, diet, feeding conditions, season, geographic area, and other variables (Lu et al., 2021; Chen and Grandison, 2017). A comparison with regional studies reveals notable variations. For instance, the fat content of Simmental cattle milk in Kazakhstan (Central Asia) ranged from 3.94% to 4.09%, with protein content at 3.22–3.39% (Khastayeva et al., 2021), values slightly higher than those observed in our study (fat: 3.54–3.93%; protein: 3.10–3.17%). Similarly, raw milk from Holstein cows in northern China exhibited lower fat (3.81–4.11%) and protein (3.02–3.20%) levels compared to our findings (Yang et al., 2013). These differences may reflect variations in cattle breeds, feeding regimes, or environmental conditions. Additionally, Ethiopian raw milk studies reported protein (3.11–3.39%), fat (3.97–4.45%), and non-fat solids (8.46–8.55%) (Tadesse and Gure, 2023), aligning closely with our results and underscoring the regional consistency in milk composition despite differing farming practices.

**Table 1:** Physicochemical characteristics of raw milk.

**Farm**	**Protein (%)**	**Fat (%)**	**Non-fat milk solids (%)**
M1	3.10	3.79	8.79
M2	3.12	3.73	8.73
M3	3.17	3.93	8.83
M4	3.11	3.54	8.77

### Diversity and abundance of ARGs and MGEs

**Figure 1:**
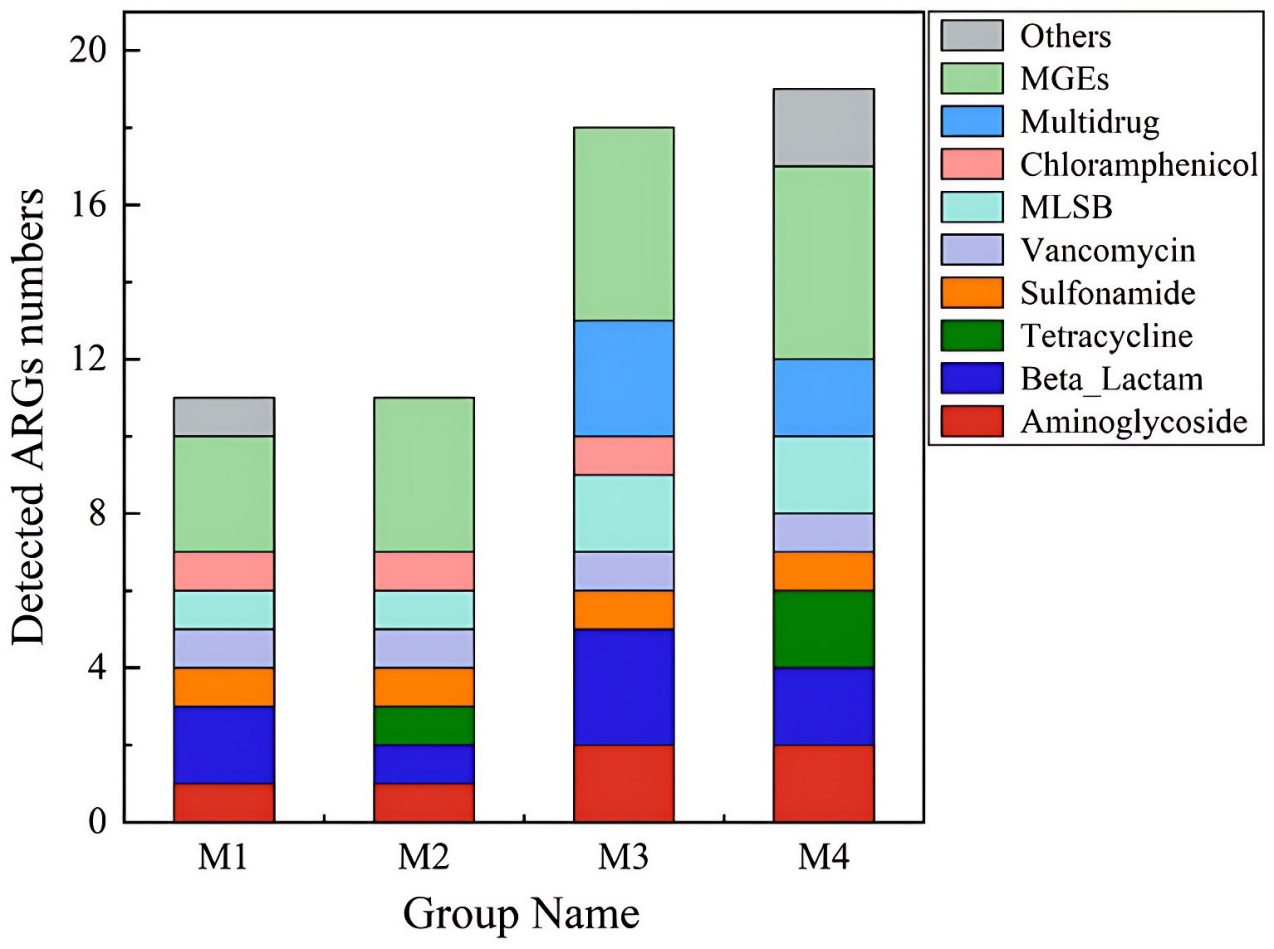
Number of ARGs and MGEs in raw milk.

**Figure 2:**
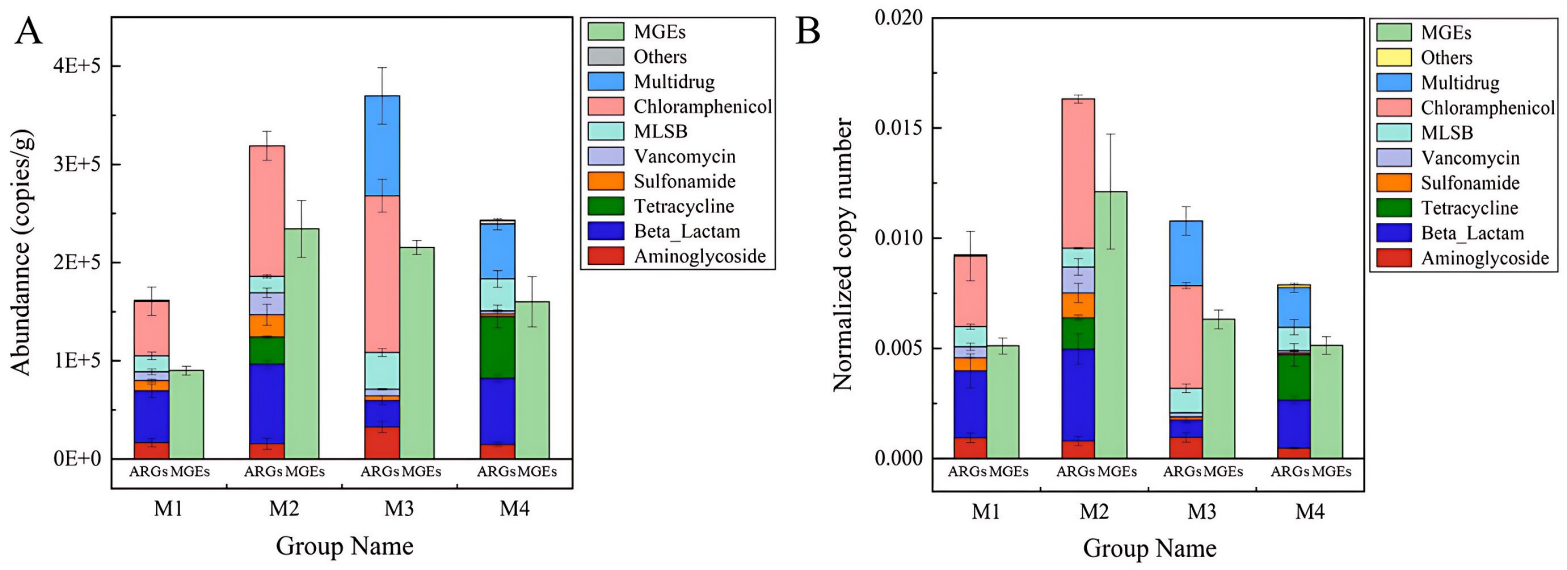
ARGs in raw milk form four farms. **(A)** Absolute copy numbers of ARGs. **(B)** The normalized copy numbers of ARGs presented as total ARG copies per bacterial cell.

In this study, ARG profiles were characterized by both gene diversity (number of unique ARG types) and absolute abundance (copies per gram of sample). A total of 31 genes, including 21 ARGs and 10 MGEs, were detected across all samples. Based on gene diversity (Figure 1), aminoglycoside resistance genes represented the highest proportion (28.57%, 6/21), followed by β*-lactam* (14.29%, 3/21) and multidrug resistance genes (14.29%, 3/21). However, qPCR quantification revealed that absolute abundance varied significantly among ARG types (Figure 2A). For example, the chloramphenicol resistance gene *cmx(A)* exhibited the highest abundance in the M3 group, despite its lower representation in gene diversity (4.76%, 1/21). This discordance highlights that gene diversity alone may not reflect environmental risks, as high-abundance ARGs are more likely to persist and transfer within microbial communities. The number of aminoglycoside and beta-lactam ARGs was higher than other types of ARGs (Figure 1). These two classes of antibiotics, known for their broad-spectrum antimicrobial efficacy, are extensively utilized in the therapeutic management of a diverse array of bacterial infections (Shoaib et al., 2024). In 2020, the total amount of antimicrobial drugs used in China was 32,776.298 tons, with domestic production accounting for 99.29% and imports accounting for 0.71%. Tetracyclines (10,002.733 tons, 30.52%), Sulfonamides and Potentiators (4,287.876 tons, 13.08%), and β-lactam and Inhibitors (4,112.629 tons, 12.55%) (Ministry of Agriculture Rural Affairs, 2020). The extensive application of these antibiotics, particularly in veterinary medicine and agriculture, could be a significant factor the observed patterns of resistance gene distribution in the dairy products. In Northwest Xinjiang, β-lactams, tetracyclines, aminoglycosides, and chloramphenicol emerge as priority antibiotics for stewardship, given their high associated ARG abundance in raw milk. Targeted reduction in their use, combined with improved farm hygiene, is vital to curbing resistance propagation in this critical dairy region.

The abundance of ARGs detected in raw milk was significant, with copy numbers ranging from 1.61 × 105 to 3.70 × 105 copies per gram of sample (Figure 2A). The adjusted abundance of ARGs per microbial cell varied between 0.0079 and 0.016, averaging 0.011 ARGs per bacterial cell (Figure 2B). The M3 group exhibited the greatest concentration of ARGs, primarily driven by chloramphenicol resistance genes (1.60 × 10^5^), multidrug resistance genes (1.02 × 10^5^), and MLSB resistance genes (3.75 × 10^4^). In addition, β-lactam, vancomycin, tetracycline resistant genes with high abundance were also detected in both raw milk samples. Here we investigated the prevalence and drivers of ARGs in raw milk from Northwest Xinjiang dairy farms. Our methodology aligns with quantitative frameworks applied in sludge composting and swine gut studies, where *Actinobacteria*-driven ARG enrichment was quantified via HT-qPCR during composting (Su et al., 2015), and feed additives were shown to reshape *Bacteroidetes/Firmicutes* ratios while amplifying ARG copies/g feces (Zhao et al., 2018). In contrast, metagenomic approaches—such as Erkorkmaz's work on dust storm-mediated ARG dispersal and Neo's characterization of riverine ARG hotspots—revealed extensive resistome diversity and HGT potential but lacked absolute quantification (Erkorkmaz et al., 2025; Neo et al., 2025). Moreover, the abundance of MGEs was highest in M2 samples (2.34 × 10^5^), followed by M3 samples (2.15 × 10^5^) (Figure 2A). The elevated presence of MGEs in these samples indicates a significant likelihood of HGT, a key process enabling the spread of ARGs within microbial communities. Notably, specific MGEs such as cIntI-1 and *tnpA* were prevalent across samples, suggesting their potential role in facilitating ARG dissemination via HGT. Integrons are known to capture and express resistance cassettes, while transposases enable gene mobility between plasmids or chromosomes.

**Figure 3:**
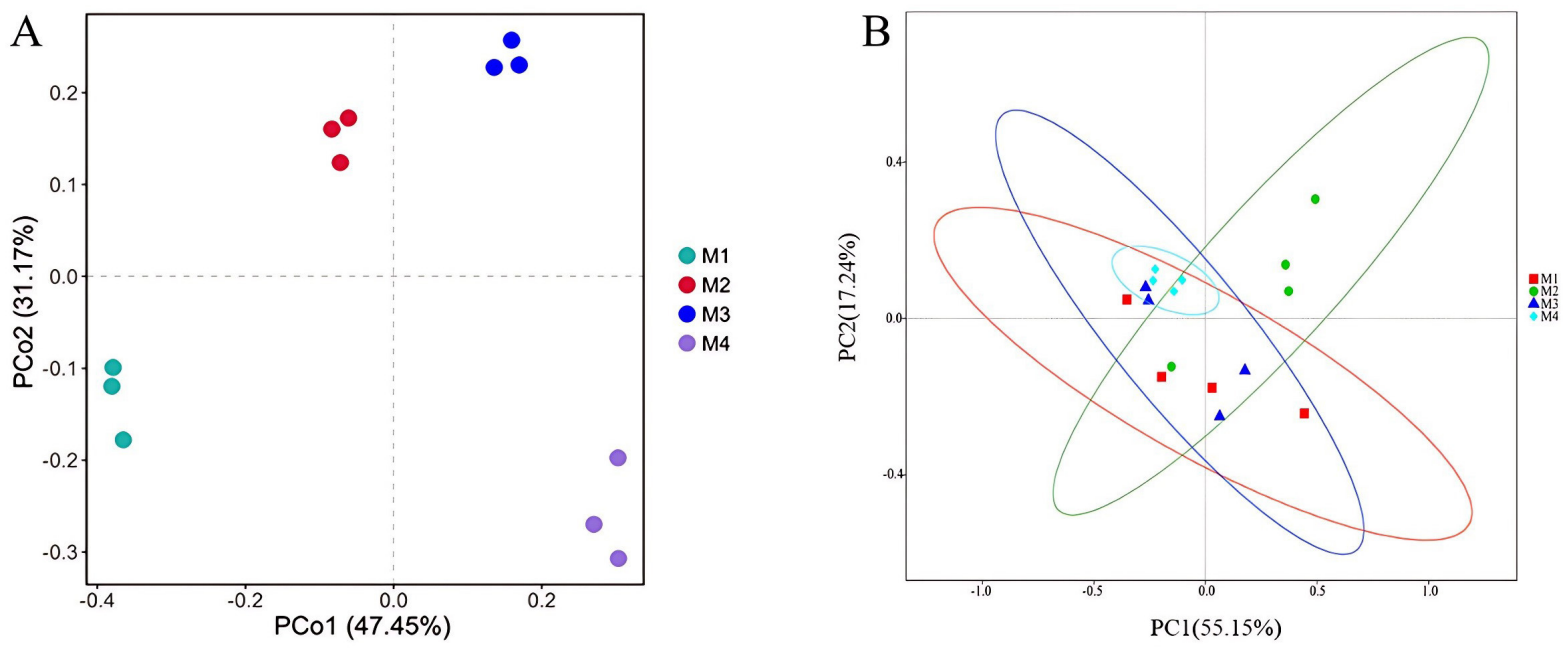
Principal coordinate analysis (PCoA) based on the Bray–Curtis distance showing the overall distribution pattern of **(A)** ARGs in raw milk; **(B)** bacterial community in raw milk.

PCoA based on Bray–Curtis distance revealed that raw milk samples from the same dairy farm clustered closely together and were distinctly separated from those of other farms. The first two principal coordinates explained 78.62% of the variation in ARGs, with PCo1 accounting for 47.45% of the total variation (Figure 3A). Four shared ARGs and one MGEs were found in all samples (Table 2), namely *blaPAO* (β-lactam), *sulA/folP-03* (sulfonamide), *tnpA-03* (transposon), *vanXD* (vancomycin), and *tnpA-03* (transposon). Distinctive ARGs were identified, with the maximum count of unique ARGs reaching 7 in the M3 group (Table 2). These results indicate differences in the composition of ARGs among four farms, which may be due to the differences in farm management levels, HGT between bacterial cells, and succession of bacterial communities (Gu et al., 2019).

**Table 2:** ARGs in four farms raw milk samples.

**Classify**	**M1**	**M2**	**M3**	**M4**
Aminoglycoside	aac-(6′)-lb-cr	aphA3-01	aadA1	aadA25
			aphA3-02	aadE
Beta_Lactam	blaPAO	blaPAO	blaCTX-M-05	blaPAO
	blaSHV-01		blaPAO	blaSHV-01
			blaSHV-01	
Tetracycline		tetQ		tetQ
				tetM-02
Sulfonamide	sulA/folP-03	sulA/folP-03	sulA/folP-03	sulA/folP-03
Vancomycin	vanXD	vanXD	vanXD	vanXD
MLSB	vatE-01	vatE-01	vatE-01	vatE-01
			vgaB-01	vgaB-01
Chloramphenicol	cmx(A)	cmx(A)	cmx(A)	
Multidrug			qacEdelta1-01	qacEdelta1-01
			yceE/mdtG-01	qacEdelta1-02
			qacEdelta1-02	
MGEs	tnpA-03	tnpA-03	tnpA-01	tnpA-01
	cIntI-1(class1)	tnpA-07	tnpA-02	tnpA-03
	IncNrep	cIntI-1(class1)	tnpA-03	cIntI-1(class1)
		IS3	tnpA-06	intl2-02
			intI-1(clinic)	IS3
Others	fosX			fosX
				qepA

### Characterization of bacterial community

From the analyzed samples, a combined total of 330,778 sequences of high quality were successfully filtered and retained. The sequence count for individual samples varied, with a minimum of 79,550 and a maximum of 85,258 sequences per sample. The high-quality sequences were grouped into 4,985 operational taxonomic units (OTUs) using a 97% similarity threshold. Using principal coordinate analysis (PCoA) with Bray–Curtis distance metrics (Figure 3B), clear clustering patterns were observed among replicate samples within each group, indicating comparable microbial community structures. At the same time, some overlap in bacterial composition was noted across different groups, suggesting partial similarities in their microbial profiles.

**Figure 4:**
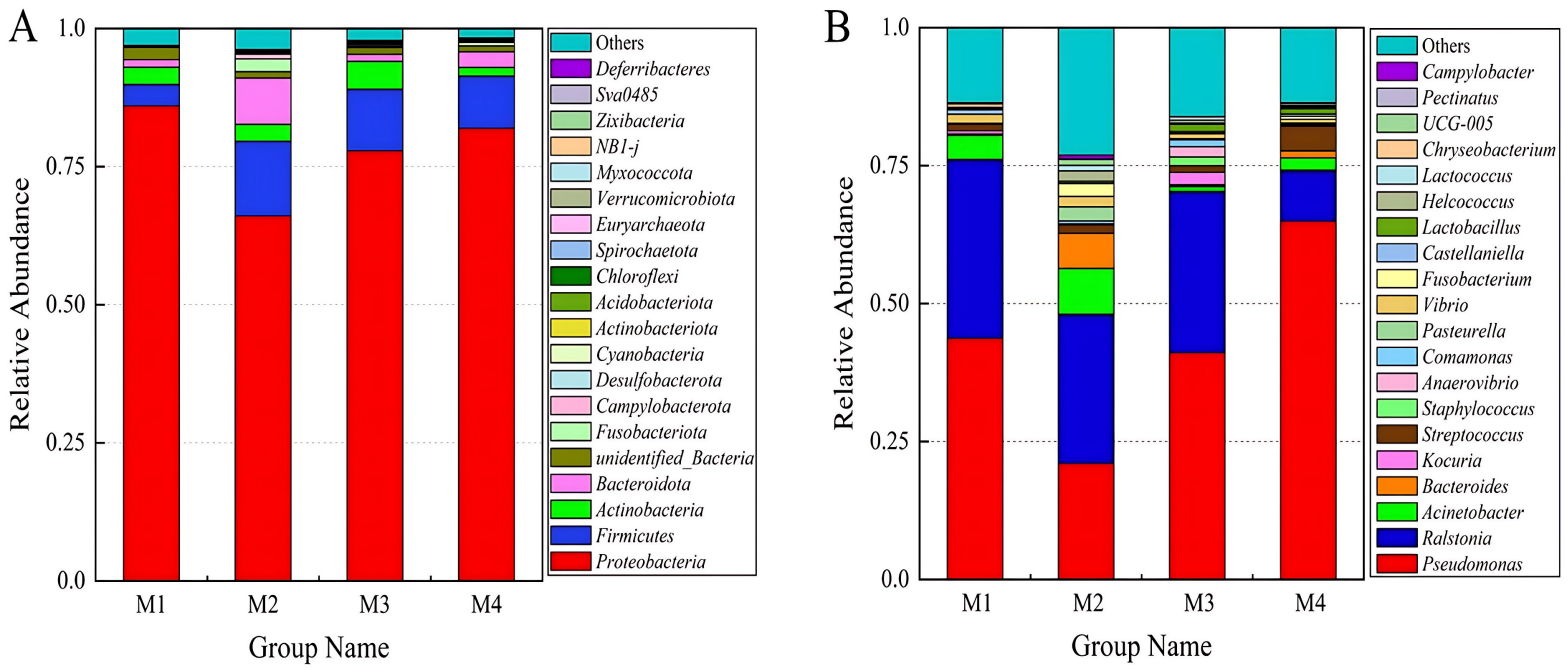
Histogram of relative species abundance, **(A)** phylum level; **(B)** genus level.

An analysis of microbial communities was conducted to evaluate their structure at both the phylum and genus levels in milk samples collected from four different dairy farms. At phylum level (Figure 4A), *Proteobacteria* was the dominant phylum in raw milk, with relative abundances up to 86.0, 66.1, 77.9, and 81.2%, respectively, followed by *Firmicutes* and *Actinobacteria*. At genus level (Figure 4B), the dominant genus was *Pseudomonas* in M1 (43.7%), M3 (41.1%) and M4 (64.9%) farms, followed by *Ralstonia*. However, in M2 farm, *Ralstonia* (27%) was the dominant genus, followed by *Pseudomonas* (21%). Both *Pseudomonas* and *Ralstonia* belong to *Proteobacteria*, gram-negative bacteria, and were conditional pathogens, *Pseudomonas* was a common cause of raw milk spoilage, and refrigerated storage may promote its growth (Vithanage et al., 2016). This dominance may be linked to Xinjiang's arid climate, which favors drought-resistant genera such as *Pseudomonas* and *Acinetobacter* within *Proteobacteria*. Similar patterns have been observed in other arid regions, where *Proteobacteria* thrive due to their adaptability to low moisture conditions (Zhao et al., 2020). The prevalence of *Ralstonia* in M2 samples may be linked to environmental sources specific to dairy farming in Xinjiang. This genus is commonly found in soil and irrigation water in agricultural regions, and its presence in raw milk could reflect contamination during milking via soil-adhered udders or biofilm formation in water pipelines. Notably, *Ralstonia* species are known to colonize dairy equipment surfaces under suboptimal sanitation conditions, further facilitating their entry into milk (Ostrov et al., 2019). The proportion of *Acinetobacter* genus was relatively high in M1 (4.4%) and M2 (8.3%). It can remain their viability in desiccated infant formula for 2 years, and to be recovered after reconstitution (Juma et al., 2016). *Chryseobacterium* were also detected, which proved have psychrotolerant and can produce proteinase, which affects the quantity of milk (Laviad-Shitrit et al., 2017). In addition, lactic acid bacteria (LAB) such as *Lactobacillus* (0.1–1.3%) and *Lactococcus* (0.09–1.0%) were also detected. The low abundance of LAB could stem from prolonged cold storage during transport, which suppresses LAB growth, or from competitive exclusion by psychrotolerant spoilage bacteria like *Pseudomonas*. The above results indicate that the bacteria with relatively high abundance in raw milk were mostly conditional pathogenic bacteria or spoilage bacteria, which may be contaminated by environmental factors such as water, soil, and air. These bacteria, particularly gram-negative pathogens like *Pseudomonas* and *Ralstonia*, are known to harbor and disseminate ARGs through HGT (Wu et al., 2020). The widespread MGEs detected in raw milk may persist through common processing steps. For instance, pasteurization eliminates viable bacteria but does not fully degrade extracellular DNA, allowing residual MGEs to potentially transfer ARGs to human gut microbiota via HGT. Studies have shown that ingested ARG-bearing DNA fragments can integrate into commensal or pathogenic bacteria in the gastrointestinal tract, compromising clinical antibiotic efficacy (Paul and Das, 2022). Thus, mitigating MGE-driven ARG dissemination in raw milk is critical to safeguarding both dairy product safety and public health. The presence of such bacteria in raw milk, combined with the high abundance of MGEs like transposons, suggests a potential reservoir for ARGs.

### Drivers of ARGs

**Figure 5:**
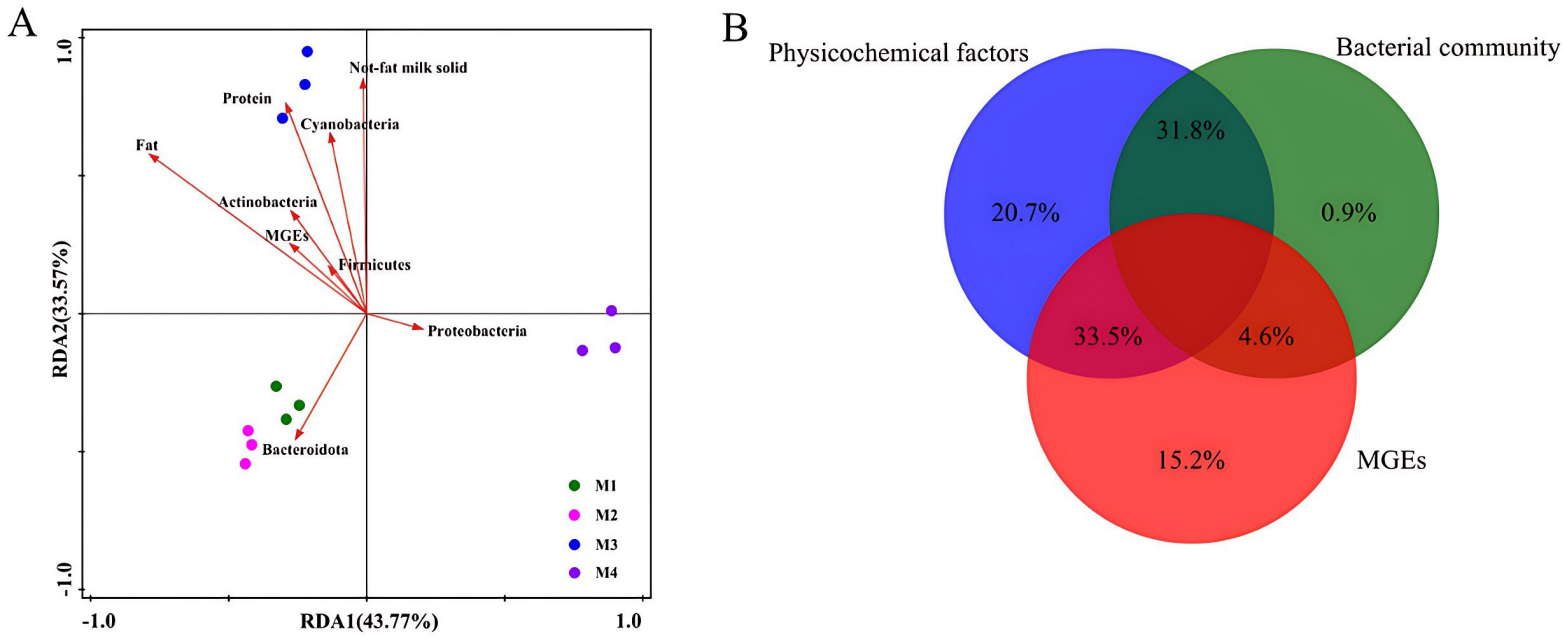
**(A)** Redundancy analysis diagram; **(B)** VPA analysis diagram.

**Figure 6:**
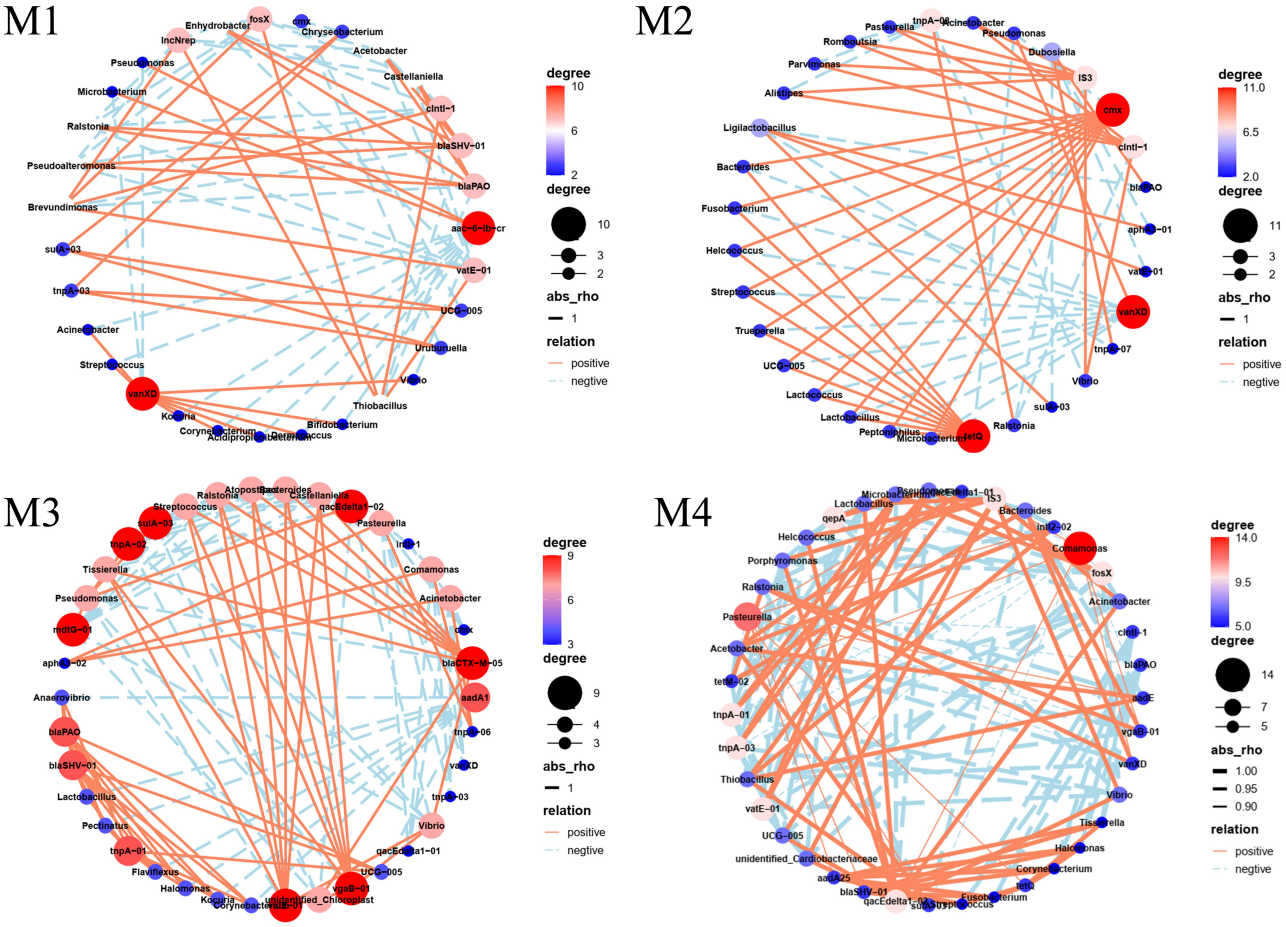
Network analysis diagram.

To systematically examine the relationships among physicochemical properties, microbial community composition, and genes associated with antibiotic resistance, redundancy analysis (RDA) was utilized as the primary analytical method. The RDA results (Figure 5A) demonstrated that physicochemical properties such as protein, fat, and non-fat milk solids showed a positive association with sample M3. Notably, M3 exhibited the highest fat content (3.93%) and non-fat milk solids (8.83%) among all samples (Table 1), which may create a nutrient-rich environment favoring bacterial taxa specialized in lipid metabolis. Additionally, *Bacteroidota* exhibited a strong positive correlation with samples M1 and M2, while Proteobacteria was closely linked to sample M4. In contrast, *Bacteroidota*, which is adept at degrading complex carbohydrates and dietary fibers (Brown and Koropatkin, 2021), correlated strongly with samples M1 and M2. This association may stem from differences in feed composition (e.g., higher fiber content in silage-based diets) or farm management practices that selectively enrich fiber-degrading taxa. VPA was conducted to distinguish (Figure 5B) the effects of physicochemical factors (20.7%), bacterial community (0.9%), and MGEs (15.2%) on ARGs. The interplay between physicochemical parameters and microbial communities contributed to 31.8% of the observed variation. In contrast, a significantly higher proportion (33.5%) was linked to the combined effects of physicochemical factors and MGEs. Procrustes analysis based on the Bray-Curtis dissimilarity matrix (Supplementary Figure S1) revealed a significant clustering pattern of ARGs detected by qPCR and bacterial 16S rRNA gene operational taxonomic units (OTUs) in raw milk samples according to the dairy farm. The goodness-of-fit test result (sum of squares M2 = 0.949, *P* < 0.0001, with 9,999 permutations) was statistically significant (Figure 6). However, the high *M*^2^ value (approaching 1) indicates substantial residual dissimilarity between the two datasets, suggesting that while the overall correlation is significant, the structural similarity between ARG distribution and microbial composition is limited. In this study, the differences in ARGs were mainly attributed to physicochemical factors, especially their interactions with bacterial communities and MGEs (31.8 and 33.5%, respectively). These interactions suggest that physicochemical factors indirectly drive the distribution and spread of ARGs by influencing bacterial communities and MGEs activity. Nevertheless, earlier research has demonstrated that MGEs, particularly *tnpA*, play a dominant role in shaping the distribution of ARGs within livestock farm wastewater systems (Xu et al., 2022). In conclusion, the predominant influences on ARGs were attributed to physicochemical elements. The observed differences in ARG composition among the four farms likely reflect variations in farm management practices. For instance, farms with suboptimal hygiene protocols—such as inadequate udder cleaning, insufficient sterilization of milking equipment, or unsanitary storage conditions—may experience heightened environmental contamination, facilitating the introduction of MGEs and ARB from soil, water, or fecal sources (Forster et al., 2022). A recent study on Chinese dairy farms demonstrated that unrestricted antibiotic usage correlated with elevated ARG abundance in raw milk, whereas farms adhering to antibiotic stewardship programs exhibited significantly reduced resistance gene loads (Liu et al., 2023). These findings underscore the critical role of stringent hygiene practices and regulated antibiotic use in mitigating ARG dissemination within dairy systems.

### Potential host bacteria of ARGs in raw milk

To investigate the potential drivers of ARGs in raw milk, a network analysis was performed to examine the connections between the 20 most prevalent bacterial genera, 21 ARGs, and 10 MGEs (Figure 6). The network analysis revealed that the count of nodes fell within a range of 30 to 38, whereas the connections, or edges, spanned from 68 to 133 across the different networks examined. In the network analysis between bacterial phyla and ARGs, the positive correlation and its strength were higher for M2 and M3, indicating that bacterial growth facilitates the dissemination of ARGs in raw milk. Variations were observed in the network analysis of ARGs and microbial communities across the four farms, with certain ARGs exhibiting the potential to associate with multiple bacterial hosts. Network analysis revealed that specific ARGs were associated with distinct bacterial hosts across the four farms. For instance, the aminoglycoside resistance gene *aac-(6*′*)-lb-cr* was linked to *Proteobacteria* and *Chloroflexi* in M1 but primarily to *Actinobacteria* and *Firmicutes* in M3. Similarly, the β-lactam resistance gene *blaPAO* exhibited host plasticity: in M1, its hosts included *Bdellovibrionota, Cyanobacteria*, and *Gemmatimonadota*, whereas in M3 and M4, it predominantly associated with *Actinobacteria, Firmicutes*, and *Euryarchaeota*. Notably, *vanXD* (vancomycin resistance) demonstrated broad host adaptability, co-occurring with *Firmicutes, Actinobacteria*, and *Bacteroidota* in M1, but shifted to *Gemmatimonadota* and *Verrucomicrobiota* in M3 and M4. These findings highlight that even conserved ARGs like *sulA/folP-03* (sulfonamide resistance) and *tnpA-03* (transposase) exhibited farm-specific host associations (e.g., *Fusobacteriota* and *Desulfobacterota* in M1 vs. *Proteobacteria* in M2). Importantly, certain ARGs were shared across farms but hosted by divergent phyla. For example, *tetQ* (tetracycline resistance) correlated with *Firmicutes* and *Actinobacteria* in M2, while in M4, it co-occurred with *Proteobacteria* and *Acidobacteriota*. This suggests that farm-specific environmental conditions or microbial niche competition may drive host flexibility. In addition, the potential hosts of the same ARGs in four farms were different, which may be due to differences in the bacterial community structure of four farms (Figure 6). Network analysis revealed significant co-occurrence patterns between β-lactam resistance genes and *Actinobacteria/Firmicutes* (Mantel *r* > 0.65, *p* < 0.01), suggesting these phyla may serve as important reservoirs for β-lactam resistance determinants. Similarly, *Firmicutes, Actinobacteria*, and *Gemmatimonadota* showed strong statistical associations with vancomycin resistance genes (Procrustes analysis, M^2^ = 0.82), though further genomic validation is needed to confirm direct host relationships. Notably, *Actinobacteria* and *Firmicutes*—common in soil and animal gut microbiomes—likely proliferated in farms with frequent β-lactam antibiotic us, exerting selective pressure that enriched these taxa as ARG reservoir. Conversely, *Euryarchaeota's* association with β-lactam resistance in M3 and M4 may reflect environmental contamination from poorly managed manure or water sources, as these methanogens thrive in anaerobic, nutrient-rich conditions (He et al., 2015). Similarly, vancomycin resistance genes were predominantly hosted by Firmicutes and Actinobacteria—Gram-positive bacteria often linked to fecal contamination—suggesting inadequate hygiene practices in M1 and M2 (Memili et al., 2022). The co-occurrence of *Gemmatimonadota* with *vanXD* in M3 and M4, however, implies soil-borne transmission, as this phylum is prevalent in agricultural soils and may infiltrate milk through dust or unsterilized equipment. These farm-specific host patterns underscore how management practices and microbial niche dynamics collectively shape ARG-host associations, necessitating tailored interventions to disrupt resistance gene dissemination. Overall, *Actinobacteria, Firmicutes* and *Euryarchaeota* were the dominant β-lactam resistance genes hosts. The dominant Vancomycin hosts belonged to the *Firmicutes, Actinobacteria, Gemmatimonadota*, all of which were Gram positive bacteria. These findings align closely with earlier research outcomes, demonstrating an agreement with prior studies (Tong et al., 2022). ARGs can increase in abundance through VGT as host bacteria proliferate. Additionally, ARGs can be disseminated to pathogenic bacteria through HGT mediated by MGEs, thereby posing a severe threat to human health. Actinobacteria, often associated with soil and environmental contamination, may indicate inadequate hygiene practices during milking or storage if present in raw milk, and their ability to produce spores that survive pasteurization poses a risk to milk quality and safety (Jose et al., 2021). The prevalence of *Firmicutes* in raw milk—a phylum that includes gut-associated genera such as *Clostridium, Staphylococcus*, and *Bacillus*—suggests potential fecal contamination due to inadequate hygiene practices during milking or storage (e.g., insufficient udder cleaning, unsterilized equipment, or improper manure management). Of particular concern are pathogenic species like *Clostridium botulinum* and *Staphylococcus aureus*, which can produce heat-resistant toxins (e.g., botulinum neurotoxin and staphylococcal enterotoxins) that may survive pasteurization, increasing the risk of foodborne illness (Walker-York-Moore et al., 2017). Additionally, *Bacillus cereus*, a common spoilage organism in dairy products, can cause diarrheal and emetic syndromes in consumers, further highlighting the public health risks associated with fecal contamination in raw milk (Cui et al., 2016). While *Gemmatimonadota* are commonly found in soil environments, their role as ARG hosts remains poorly characterized in current literature. Limited studies suggest their potential involvement in ARG dissemination through soil-water systems, but conclusive evidence linking *Gemmatimonadota* to clinically relevant resistance transfer is currently lacking (Zhang et al., 2023). Among the detected ARGs, β-lactam resistance genes and vancomycin resistance genes pose the most significant public health risks. β-lactamases are clinically critical due to their role in conferring resistance to penicillins and cephalosporins—cornerstone antibiotics in human medicine—and their frequent association with MGEs, which facilitates rapid dissemination across bacterial species (Agarwal et al., 2023). Similarly, vancomycin resistance genes threaten the efficacy of a last-resort antibiotic for treating multidrug-resistant Gram-positive infection. These ARGs were not only highly abundant in raw milk but also linked to clinically relevant pathogens detected in this study (Haas et al., 2023). Future research could build upon our network-based host predictions by employing metagenome-assembled genomes (MAGs) to resolve complete microbial genomes from raw milk samples. Such approaches would further clarify associations between dominant taxa and specific ARGs, enabling precise validation of host-pathogen relationships and horizontal transfer mechanisms.

## Conclusions

The prevalence and dissemination of ARGs in raw milk from dairy farms in the northwestern region of Xinjiang were analyzed. High throughput qPCR sequencing showed significant abundance of β-lactam, tetracycline, aminoglycoside and chloramphenicol. These analytical methods-−16S rRNA sequencing, network analysis, and VPA—collectively aimed to identify the key drivers and hosts of ARG dissemination in raw milk. Based on the existing data, it is speculated that *Actinobacteria* and *Firmicutes* may be the main potential hosts of β-lactam resistance genes. The *Firmicutes, Actinobacteria* and *Gemmatimonadota* phyla may dominate the host distribution of vancomycin resistance genes. These findings advocate for Xinjiang-specific regulations, including mandatory ARG monitoring in raw milk and training programs for farmers on antibiotic stewardship and hygiene practices. In conclusion, this study highlights the need to improve farm management practices, including avoiding the use of antibiotics such as β-lactam, tetracycline, aminoglycoside, and chloramphenicol associated with the detected ARGs. In addition, by controlling physical and chemical factors such as feed composition, temperature and humidity, as well as strengthening hygiene measures, dairy farms can mitigate the spread of ARGs in raw milk.

## Data Availability

The original contributions presented in the study are included in the article/Supplementary material, further inquiries can be directed to the corresponding author.
