# Supplementary figures and images for: Prevalence of antibiotic resistance genes its association with microbiota in raw milk of northwest Xinjiang
Source: Front Microbiol. 2025 Jul 11;16:1595051. doi: 10.3389/fmicb.2025.1595051 (PMC12289685; doi:10.3389/fmicb.2025.1595051)

Supporting Information


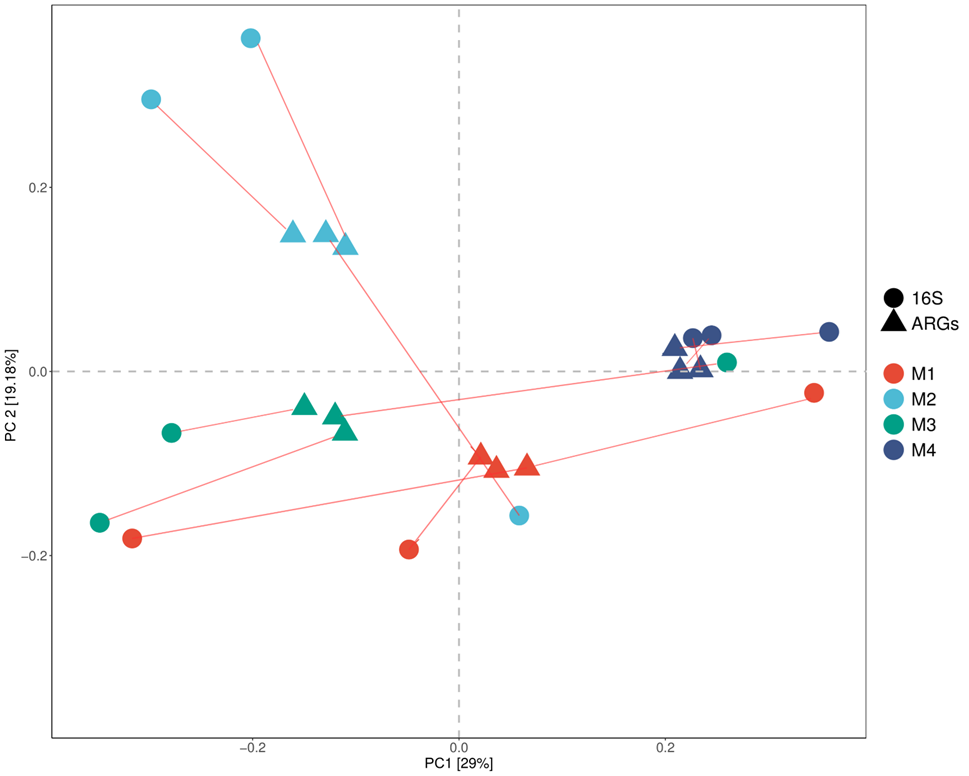


Fig. S1. Procrustes analysis diagram.

Supplement: Supplementary file 1 [file Table_1.docx]
