# Supplementary material for: Prevalence of antibiotic resistance genes its association with microbiota in raw milk of northwest Xinjiang
Source: Front Microbiol. 2025 Jul 11;16:1595051. doi: 10.3389/fmicb.2025.1595051 (PMC12289685; doi:10.3389/fmicb.2025.1595051)
Supplement: Supplementary file 2 [file Table_2.docx]

Table 1. Physicochemical characteristics of raw milk.

| Farm | Protein (%) | Fat (%) | Non-fat milk solids (%) |
| --- | --- | --- | --- |
| M1 | 3.10 | 3.79 | 8.79 |
| M2 | 3.12 | 3.73 | 8.73 |
| M3 | 3.17 | 3.93 | 8.83 |
| M4 | 3.11 | 3.54 | 8.77 |

Table 2. ARGs in four farms raw milk samples.

| Classify | M1 | M2 | M3 | M4 |
| --- | --- | --- | --- | --- |
| Aminoglycoside | aac-(6′)-lb-cr | aphA3-01 | aadA1 | aadA25 |
|  |  |  | aphA3-02 | aadE |
| Beta_Lactam | blaPAO | blaPAO | blaCTX-M-05 | blaPAO |
|  | blaSHV-01 |  | blaPAO | blaSHV-01 |
|  |  |  | blaSHV-01 |  |
| Tetracycline |  | tetQ |  | tetQ |
|  |  |  |  | tetM-02 |
| Sulfonamide | sulA/folP-03 | sulA/folP-03 | sulA/folP-03 | sulA/folP-03 |
| Vancomycin | vanXD | vanXD | vanXD | vanXD |
| MLSB | vatE-01 | vatE-01 | vatE-01 | vatE-01 |
|  |  |  | vgaB-01 | vgaB-01 |
| Chloramphenicol | cmx(A) | cmx(A) | cmx(A) |  |
| Multidrug |  |  | qacEdelta1-01 | qacEdelta1-01 |
|  |  |  | yceE/mdtG-01 | qacEdelta1-02 |
|  |  |  | qacEdelta1-02 |  |
| MGEs | tnpA-03 | tnpA-03 | tnpA-01 | tnpA-01 |
|  | cIntI-1(class1) | tnpA-07 | tnpA-02 | tnpA-03 |
|  | IncNrep | cIntI-1(class1) | tnpA-03 | cIntI-1(class1) |
|  |  | IS3 | tnpA-06 | intl2-02 |
|  |  |  | intI-1(clinic) | IS3 |
| Others | fosX |  |  | fosX |
|  |  |  |  | qepA |
