# Supplementary material for: Prevalence of antibiotic resistance genes its association with microbiota in raw milk of northwest Xinjiang
Source: Front Microbiol. 2025 Jul 11;16:1595051. doi: 10.3389/fmicb.2025.1595051 (PMC12289685; doi:10.3389/fmicb.2025.1595051)
Supplement: Supplementary file 9 [file Table_9.docx]

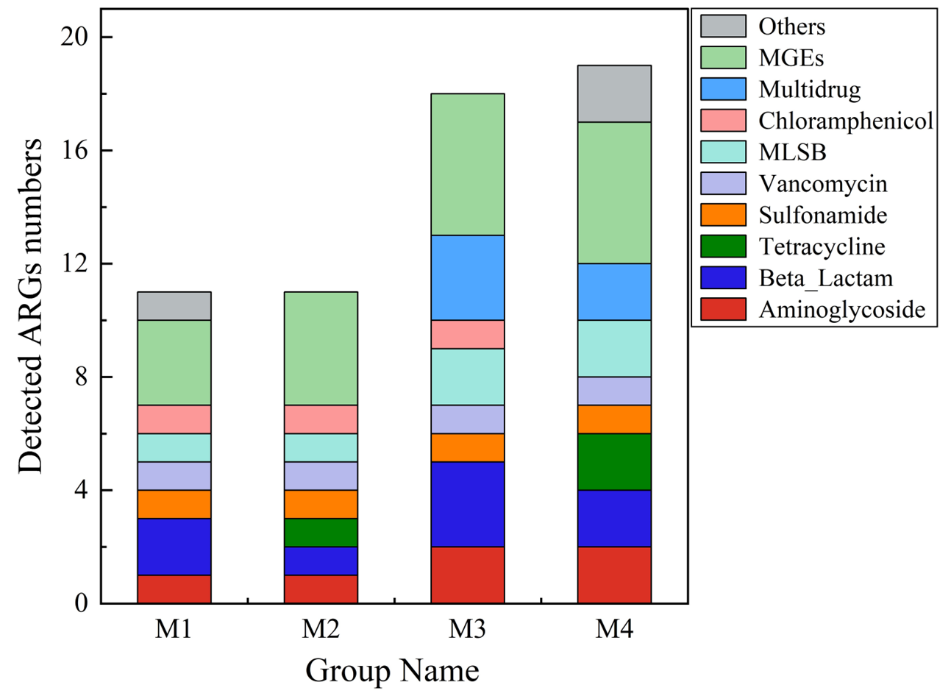


Fig. 1. Number of ARGs and MGEs in raw milk


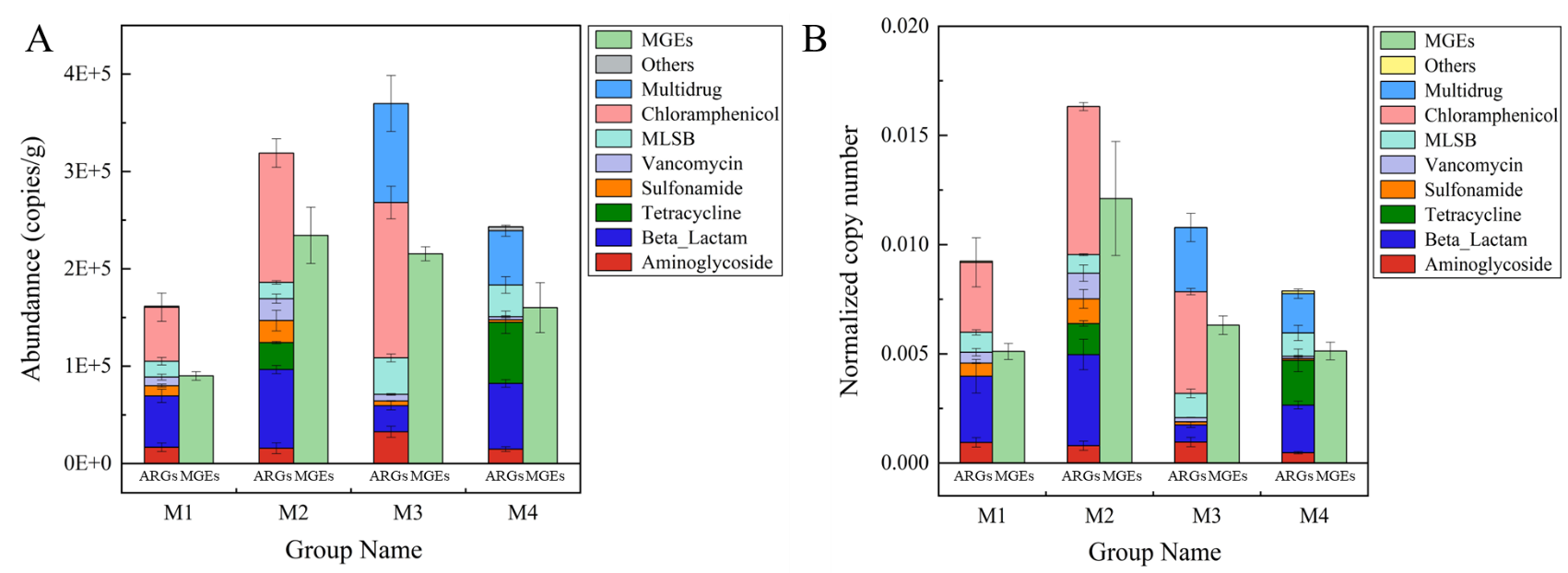
Fig. 2. ARGs in raw milk form four farms. (A) Absolute copy numbers of ARGs. (B) the normalized copy numbers of ARGs presented as total ARG copies per bacterial cell.


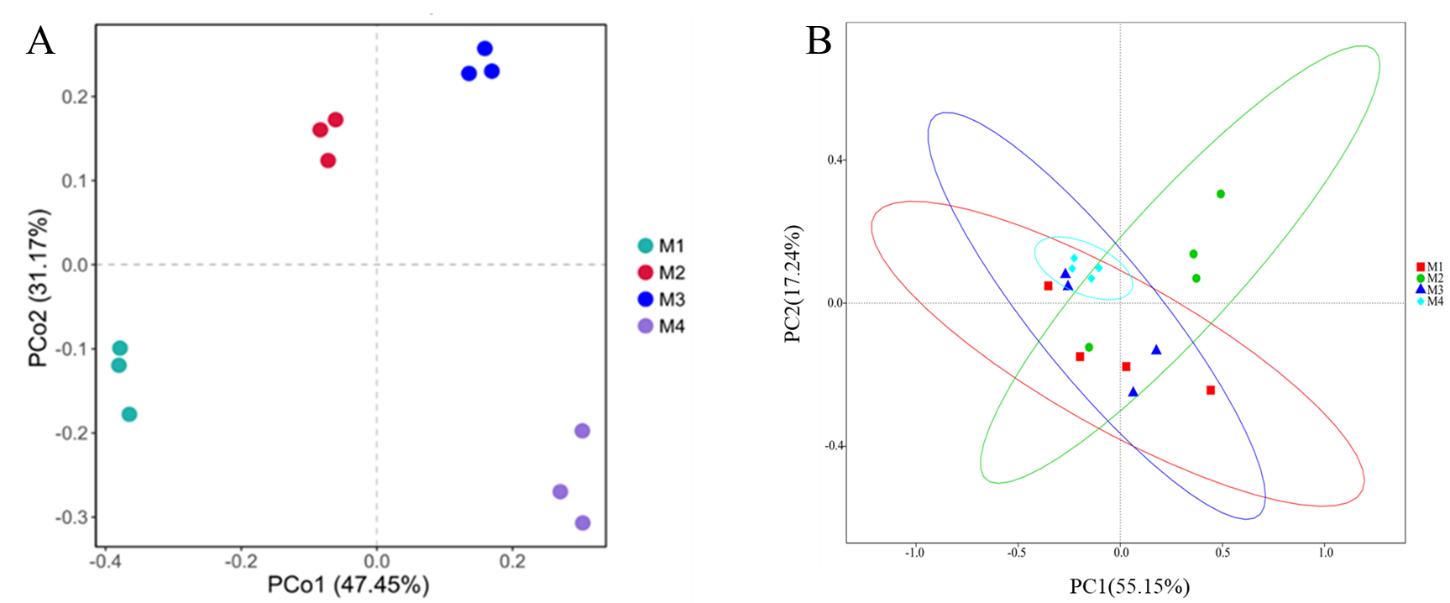


Fig. 3. Principal coordinate analysis (PCoA) based on the Bray−Curtis distance showing the overall distribution pattern of (A) ARGs in raw milk; (B) bacterial community in raw milk.


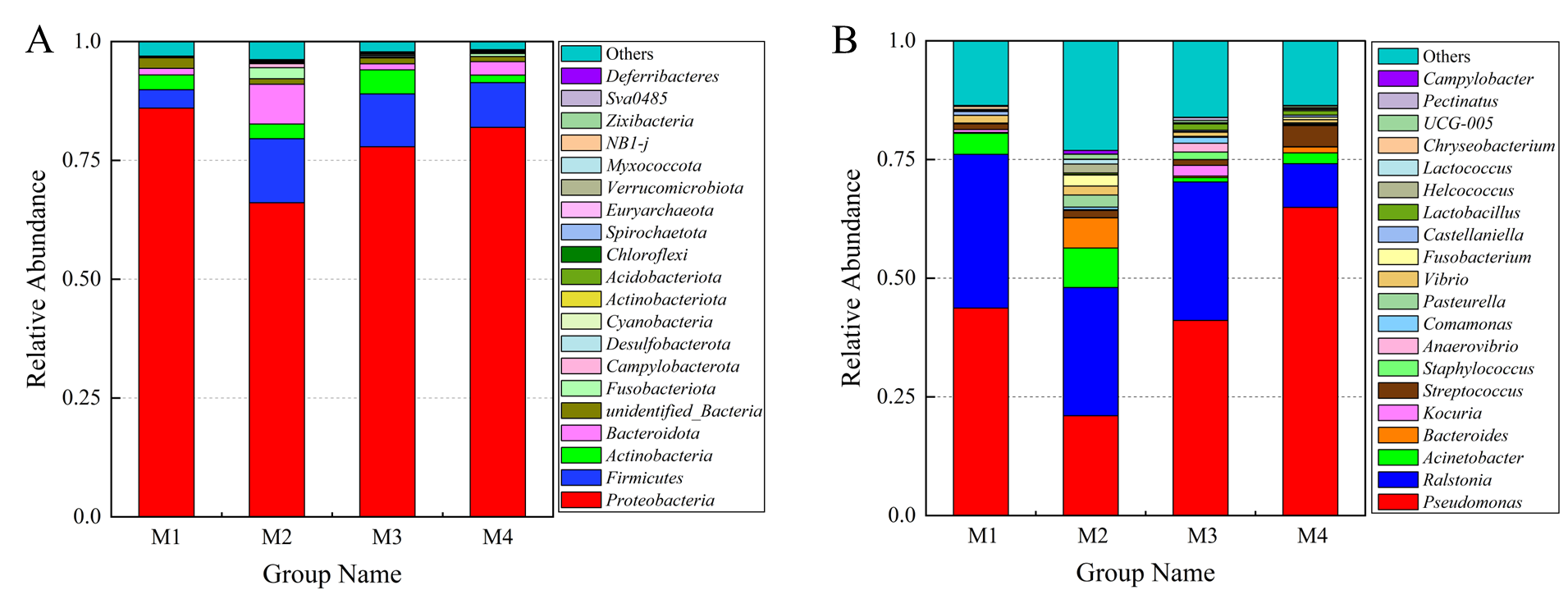


Fig. 4. Histogram of relative species abundance, (A) phylum level; (B) genus level


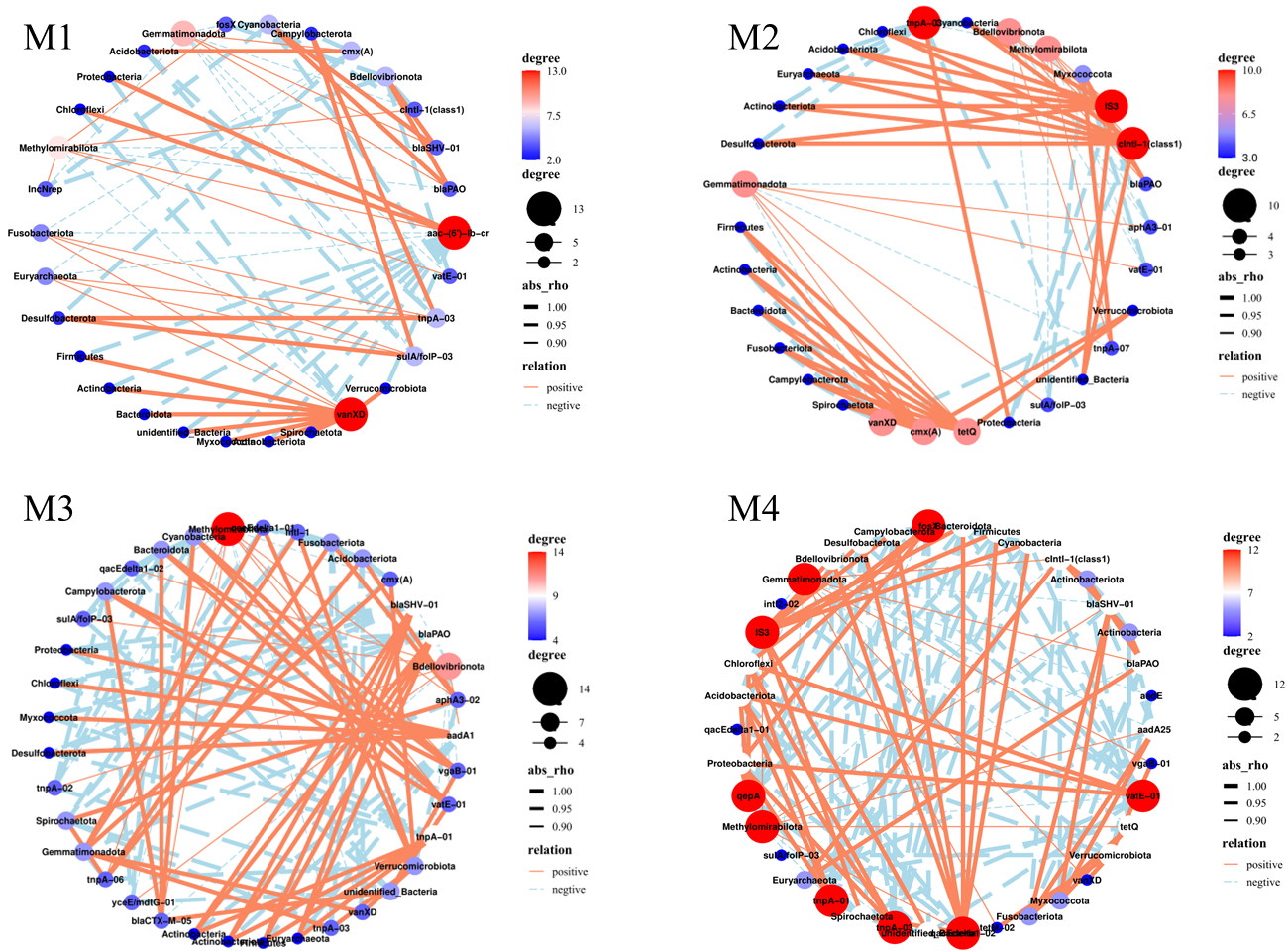


Fig. 5. Network analysis diagram


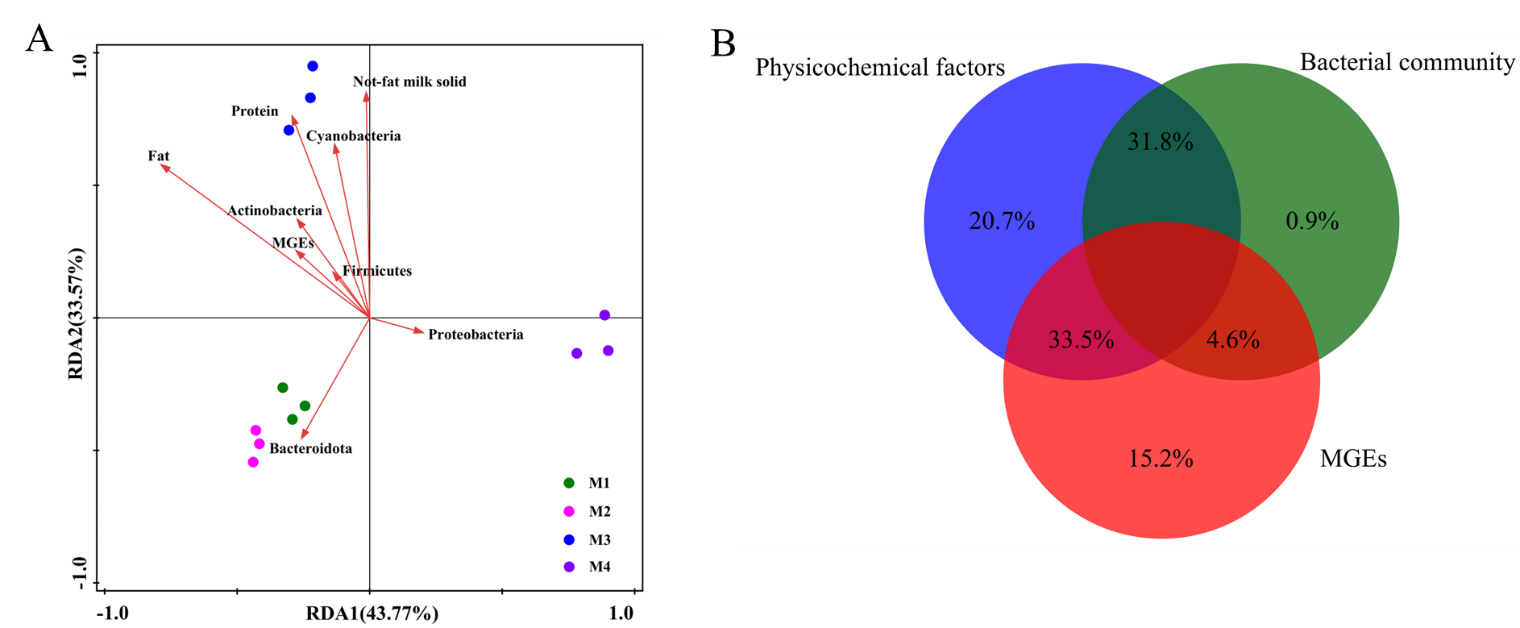


Fig. 6. (A) Redundancy analysis diagram; (B) VPA analysis diagram
