# Supplementary material for: Prevalence of antibiotic resistance genes its association with microbiota in raw milk of northwest Xinjiang
Source: Front Microbiol. 2025 Jul 11;16:1595051. doi: 10.3389/fmicb.2025.1595051 (PMC12289685; doi:10.3389/fmicb.2025.1595051)
Supplement: Supplementary file 10 [file Presentation_1.pptx]

## Slide 1
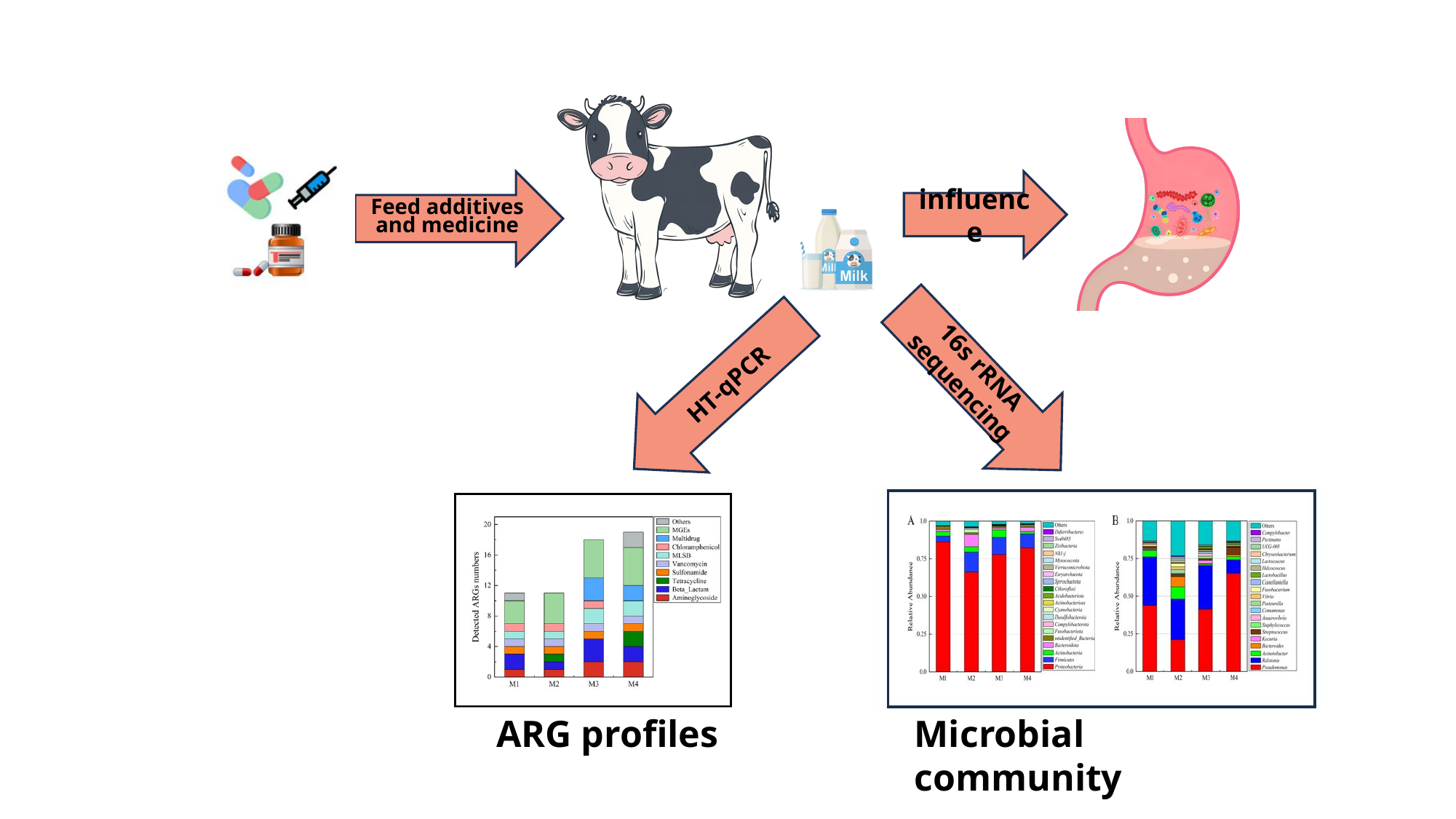

Feed additives and medicine
influence
16s rRNA sequencing
HT-qPCR
ARG profiles
Microbial community
